# Ecosystem Resilience and Limitations Revealed by Soil Bacterial Community Dynamics in a Bark Beetle-Impacted Forest

**DOI:** 10.1128/mBio.01305-17

**Published:** 2017-12-05

**Authors:** Kristin M. Mikkelson, Brent M. Brouillard, Chelsea M. Bokman, Jonathan O. Sharp

**Affiliations:** a Department of Civil and Environmental Engineering, Colorado School of Mines, Golden, Colorado, USA; b Hydrologic Science and Engineering Program, Colorado School of Mines, Golden, Colorado, USA; CEH-Oxford

**Keywords:** 16S DNA, 16S RNA, biogeochemistry, forest mortality, microbial ecology, threshold

## Abstract

Forested ecosystems throughout the world are experiencing increases in the incidence and magnitude of insect-induced tree mortality with large ecologic ramifications. Interestingly, correlations between water quality and the extent of tree mortality in Colorado montane ecosystems suggest compensatory effects from adjacent live vegetation that mute responses in less severely impacted forests. To this end, we investigated whether the composition of the soil bacterial community and associated functionality beneath beetle-killed lodgepole pine was influenced by the extent of surrounding tree mortality. The most pronounced changes were observed in the potentially active bacterial community, where alpha diversity increased in concert with surrounding tree mortality until mortality exceeded a tipping point of ~30 to 40%, after which diversity stabilized and decreased. Community structure also clustered in association with the extent of surrounding tree mortality with compositional trends best explained by differences in NH_4_^+^ concentrations and C/N ratios. C/N ratios, which were lower in soils under beetle-killed trees, further correlated with the relative abundance of putative nitrifiers and exoenzyme activity. Collectively, the response of soil microorganisms that drive heterotrophic respiration and decay supports observations of broader macroscale threshold effects on water quality in heavily infested forests and could be utilized as a predictive mechanism during analogous ecosystem disruptions.

## IMPORTANCE

Forests around the world are succumbing to insect infestation with repercussions for local soil biogeochemistry and downstream water quality and quantity. This study utilized microbial community dynamics to address why we are observing watershed scale biogeochemical impacts from forest mortality in some impacted areas but not others. Through a unique “tree-centric” approach, we were able to delineate plots with various tree mortality levels within the same watershed to see if surviving surrounding vegetation altered microbial and biogeochemical responses. Our results suggest that forests with lower overall tree mortality levels are able to maintain “normal” ecosystem function, as the bacterial community appears resistant to tree death. However, surrounding tree mortality influences this mitigating effect with various linear and threshold responses whereupon the bacterial community and its function are altered. Our study lends insight into how microscale responses propagate upward into larger-scale observations, which may be useful for future predictions during analogous disruptions.

## INTRODUCTION

Ongoing climate change poses threats to temperature- and water-sensitive ecosystems such as forests, with far-reaching implications for water quality and greenhouse gas exchange (1, 2). Montane forested regions, in particular, have experienced higher temperatures and drought stress, resulting in greater susceptibility to beetle infestation and unprecedented tree mortality (3). The management of these areas has become a primary concern (4), with accurate predictions needed to assess the level at which intervention is necessary. Despite the impact these perturbed forests have on human health and well-being, we still do not fully understand why some beetle-killed forests elicit a strong hydrologic or biogeochemical response (2, 5–8) while others do not (9, 10). Tree mortality on this scale has the capacity to shift a forest from a carbon sink to a source (1) or adversely alter the quality of water we consume (2, 11). Hence, we need to better understand the ecosystem response from the watershed scale down to the microscale to enable predictions of when and where detrimental impacts will occur. Microorganisms contribute to changes in greenhouse gas flux through heterotrophic respiration (12) and regulate biogeochemical cycling (13), but communities also have the ability to resist changes resulting from these perturbations (14), potentially muting ecosystem responses after a large-scale disturbance.

Unlike more-studied forms of forest disruption such as logging and fire, beetle-induced tree mortality presents a unique interplay between changing hydrologic and biogeochemical regimes (15). When a forest succumbs to beetle infestation, canopy and litter deposition are altered and transpiration ceases. This leads to changes in local hydrology as canopy interception decreases, water uptake through the roots ceases, and radiation penetration increases. Shifts in terrestrial C and N cycling concurrently occur, as root uptake of nutrients ceases and woody debris degradation increases (16). Terrestrial microorganisms play a large role in localized biogeochemical cycling through assimilation and decay processes with mobilization as gaseous and aqueous species (17). As these and other physicochemical parameters change during ecosystem disturbance, soil microorganisms can adapt, which in turn affects C and N cycling (18). Therefore, it is logical that there would be distinct microbial signatures beneath beetle-impacted trees in association with these changes. Fungal communities have shown extensive changes after beetle-induced tree mortality, with decreases in fungi associated symbiotically with tree roots coupled with increases in saprotrophic fungi (19). Surprisingly, studies have not reported a similar magnitude of response of the bacterial community after insect infestation, as observations have varied in magnitude (7, 9, 19, 20). This suggests that the local bacterial soil community within these perturbed forests might have an inherent level of resilience to this type of ecosystem disruption.

While studies have investigated how the microbial community changes beneath beetle-killed trees (7, 9, 19, 20), little is understood about why the magnitude of these changes differs between studies. Recently, it has been determined that a certain threshold of localized tree mortality must be attained before observable changes in soil carbon and nitrogen pools and respiration rates beneath beetle-killed lodgepole pine trees occur (21), presumably because adjacent live trees exert a compensatory effect following a forest disturbance. Analogously, observed impacts on ecosystem processes and resident soil microbial communities during forest harvesting have occurred only after compaction exceeded a certain level (22) and can be witnessed decades after the disturbance (23).

In building upon these collective insights, we hypothesized that the soil bacterial community beneath beetle-killed trees remains stable until surrounding tree mortality (STM) exceeds a threshold after which there is an ecologic adaptation to the perturbed soil environment. To this end, we sampled soils under both live and beetle-killed lodgepole pine (*Pinus contorta*) trees in tree-centric plots containing various tree mortality levels. We utilized 16S rRNA and rRNA gene sequencing along with biogeochemical analyses (21) and functional exoenzyme activity assays to determine whether or not changes in community composition and functionality were linked to plot scale tree mortality levels.

## RESULTS

### Relationship between the soil bacterial community and STM.

**FIG 1:**
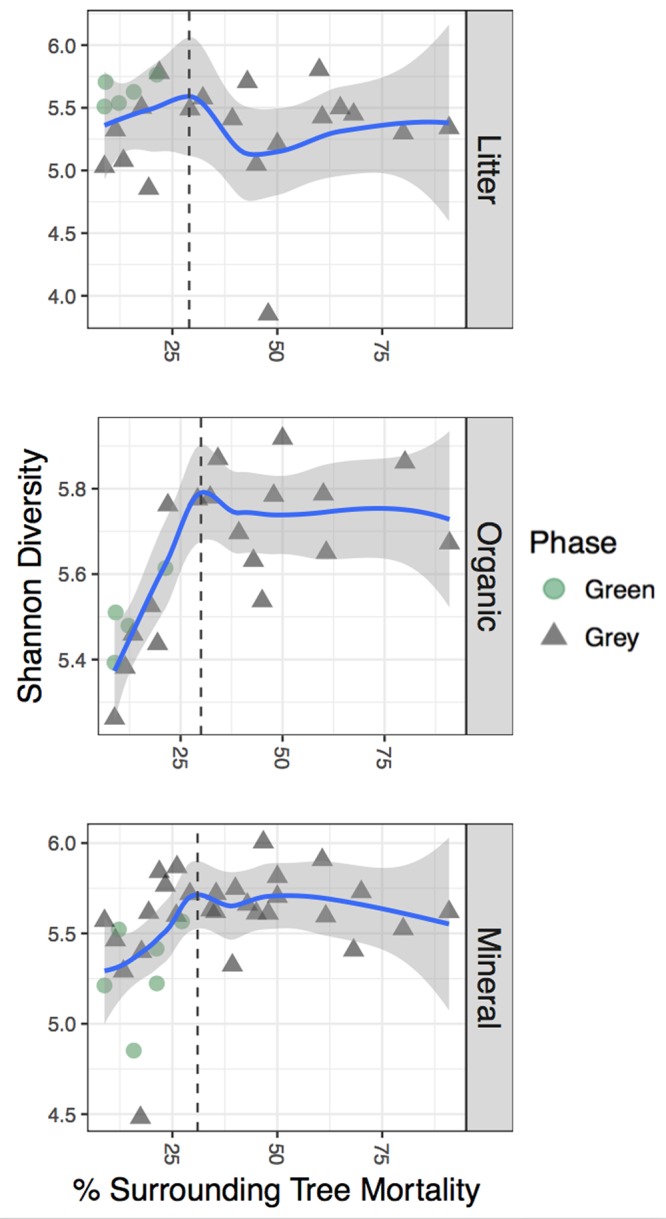
Alpha diversity measured by using the Shannon diversity metric of the RNA community increases with the extent of STM surpassing a threshold level of STM. The shaded regions around the LOESS-smoothed regression represent ±1 standard error, and shape indicates tree phase, with triangles representing gray phase and circles representing green phase. Vertical dotted lines are drawn at approximate breakpoints.

To determine whether the terrestrial microbial community was influenced by the extent of STM, we sampled three near-surface soil horizons beneath beetle-impacted (gray phase) trees in tree-centric plots containing various degrees of STM. Microbial signatures of both the RNA and DNA amplicon profiles (referred to here as the RNA and DNA communities, respectively) were then compared between soils under live green phase trees and dead gray phase trees. The strongest shifts in alpha diversity, measured as Shannon diversity (Fig. 1) and community richness (see Fig. S1 in the supplemental material), were found in the RNA community within the organic and mineral layers. Diversity measured in conjunction with the extent of STM significantly increased (Spearman correlation and Shannon diversity index: organic, *P* = 0.000 and rho = 0.95; mineral, *P* = 0.019 and rho = 0.709; observed organic, *P* = 0.017 and rho = 0.783; mineral, *P* = 0.011 and rho = 0.729) until a tipping point where it stabilized even as STM continued to increase. This threshold level ranged from 30 to 50% STM, depending on the metric studied. On the basis of these findings in conjunction with an analogous observed change in nitrogen speciation after STM surpassed approximately 40% (21), we subsequently binned the samples into high impact (>40% STM) and low impact (<40% STM) for further analyses. We found that trends within the RNA community were more significant than those in the DNA community (Fig. S2 and S3), with greater diversity under beetle-killed trees surrounded by high mortality levels. Interestingly, these trends were most pronounced in the mineral layer, with occasional significance in the organic layer.

10.1128/mBio.01305-17.1 FIG S1  Alpha diversity, measured as observed RSVs of the RNA community, increases with the extent of STM surpassing a threshold level of STM. The shaded regions around the LOESS-smoothed regression represent ±1 standard error, and shape indicates tree phase, with triangles representing gray phase and circles representing green phase. Vertical dotted lines are drawn at approximate breakpoints. Download FIG S1, TIF file, 0.6 MB. Copyright © 2017 Mikkelson et al. 2017 Mikkelson et al. This content is distributed under the terms of the Creative Commons Attribution 4.0 International license.

10.1128/mBio.01305-17.2 FIG S2  Alpha diversity under low-impact, high-impact, and green phase trees of the RNA and DNA communities in the litter, organic, and mineral soil layers. Significantly different diversity measurements under gray phase compared to green phase are indicated by a *P* value of <0.05. Points are indicative of outliers. Download FIG S2, PDF file, 0.1 MB. Copyright © 2017 Mikkelson et al. 2017 Mikkelson et al. This content is distributed under the terms of the Creative Commons Attribution 4.0 International license.

10.1128/mBio.01305-17.3 FIG S3  Alpha diversity of the DNA community increases with the extent of STM until a threshold level. The shaded regions around the LOESS-smoothed regression represent ±1 standard error, and shape indicates tree phase. Vertical dotted lines are drawn at approximate breakpoints. Download FIG S3, PDF file, 0.05 MB. Copyright © 2017 Mikkelson et al. 2017 Mikkelson et al. This content is distributed under the terms of the Creative Commons Attribution 4.0 International license.

In addition, the ecologic signatures of both RNA and DNA communities displayed a significant relationship with the extent of STM when assessed by using UniFrac beta diversity measures (Fig. S4 and S5). In building upon the hypothesized STM threshold, community composition separately clustered into those under high-impact, low-impact, and green phase trees (Fig. S4 and S5). Similar to alpha diversity, significant trends were more commonly found in the organic (Adonis unweighted UniFrac: RNA, *P* = 0.007 and *R*^2^ = 0.126; DNA, *P* = 0.002 and *R*^2^ = 0.131) and mineral (Adonis unweighted UniFrac: RNA, *P* = 0.001 and *R*^2^ = 0.089; DNA, *P* = 0.001 and *R*^2^ = 0.092) horizons, in contrast to the litter horizon. Interestingly, RNA and DNA communities both showed stronger clustering when we used the unweighted UniFrac matrix (Fig. S5), which only takes the presence or absence of ribosomal sequence variants (RSVs) into account, unlike the weighted UniFrac matrix, which takes abundances into account.

10.1128/mBio.01305-17.4 FIG S4  RNA and DNA microbial communities cluster in relation to the extent of STM. The principal-component analysis is based on the weighted UniFrac matrix with *P* values listed for significant Adonis tests. Download FIG S4, PDF file, 0.1 MB. Copyright © 2017 Mikkelson et al. 2017 Mikkelson et al. This content is distributed under the terms of the Creative Commons Attribution 4.0 International license.

10.1128/mBio.01305-17.5 FIG S5  RNA and DNA microbial communities cluster in relation to the extent of STM. The principal-component analysis is based on the unweighted UniFrac matrix with *P* values listed for significant Adonis tests. Download FIG S5, PDF file, 0.1 MB. Copyright © 2017 Mikkelson et al. 2017 Mikkelson et al. This content is distributed under the terms of the Creative Commons Attribution 4.0 International license.

**FIG 2:**
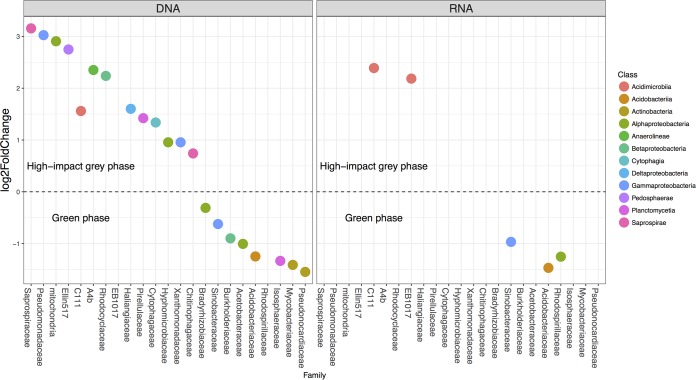
Bacterial families that are significantly different in abundance under green phase trees and high-impact gray phase trees. The color indicates the phylogenetic class the family belongs to with significant differential abundance determined when the *P* value is <0.05.

To further explore these trends, we focused on the mineral horizon, as this was where the most significant diversity trends and strongest biogeochemical responses were observed (21). Significant differences within bacterial communities under high-impact gray phase trees and those under green phase trees were best explained by the differential abundance of a comparatively modest number of microbial families (Fig. 2). Interestingly, we found a greater proportion of cladal differences at the family level in the DNA community than in the RNA community. This translated to 21 DNA families (and 5 RNA families) that significantly differed in abundance between tree phases. The clades responsible for the differences in community composition between high-impact gray and green phase trees were from similar phylogenetic origins in both the DNA and RNA communities. For instance, soil under green phase trees contained significantly more members of the families *Acidobacteriaceae* and *Sinobacteraceae*, regardless of the nucleic acid source, than soil samples from under high-STM gray phase trees. In contrast, soil samples under high-impact gray phase trees contained significantly more organisms from the *Acidimicrobiia*, “*Pedosphaerae*,” and “*Planctomycetia*” classes than did soils under green phase trees.

### Biogeochemical and microbial responses are coupled.

**FIG 3:**
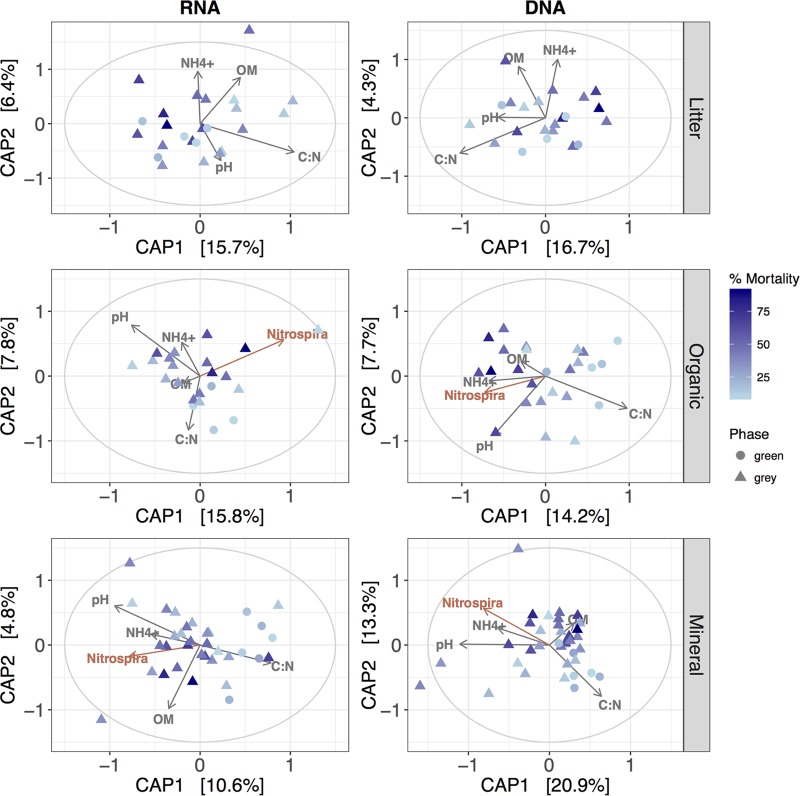
Microbial communities respond across a continuum of forest mortality levels. Clustering visualized by using CAP with the magnitude and direction of each vector representing the edaphic parameter (gray) or the taxal (red) contribution to the two principal components associated with variance observed between sampled communities.

Microbial and biogeochemical responses to ecosystem perturbation are often observed in concert (24). Therefore, we investigated how edaphic parameters in this beetle-infested forest associated with community shifts (Fig. 3). Recent findings within the near-surface soil layers in this beetle-killed watershed revealed altered C and N cycling in association with the level of STM, with the most significant shifts occurring in the mineral horizon (21). In complement to those findings, we found soil NH_4_^+^ concentrations and C/N ratios (the proportion of organic carbon to total nitrogen) strongly associated with community shifts (Fig. 3; Table S1). For instance, soils under green phase trees had higher C/N ratios, lower NH_4_^+^ concentrations, and lower pHs than both low- and high-impact gray phase trees (Fig. 3). In turn, these shifts were intertwined with altered community structure. Surprisingly, we did not find stronger correlations for the “rare” community (Table S2), despite previous evidence suggesting that the rare biosphere responds more readily to perturbed ecosystems (20).

10.1128/mBio.01305-17.7 TABLE S1  Edaphic variables explain community composition. Results of the Mantel test run by using the weighted and unweighted UniFrac matrices to indicate which edaphic parameters explain community composition. Mantel’s r is displayed with significant (*P* < 0.05) values highlighted in red. WC, water content; OM, percent organic matter; DOC, dissolved organic carbon concentration; SUVA, specific UV absorbance; TN, total nitrogen; NO_3_, nitrate concentration; NH_4_, ammonium concentration; C/N, carbon-to-nitrogen ratio. Download TABLE S1, PDF file, 0.02 MB. Copyright © 2017 Mikkelson et al. 2017 Mikkelson et al. This content is distributed under the terms of the Creative Commons Attribution 4.0 International license.

10.1128/mBio.01305-17.8 TABLE S2  Edaphic variables explain community composition in rare (A) versus abundant (B) taxa. Rare taxa were defined as the bottom 80% of taxa based on DNA relative abundance rank. Mantel’s r is displayed with significant (*P* < 0.05) values highlighted in red. WC, water content; OM, percent organic matter; DOC, dissolved organic carbon concentration; SUVA, specific UV absorbance; TN, total nitrogen; NO_3_, nitrate concentration; NH_4_, ammonium concentration; C/N, carbon-to-nitrogen ratio. Download TABLE S2, PDF file, 0.01 MB. Copyright © 2017 Mikkelson et al. 2017 Mikkelson et al. This content is distributed under the terms of the Creative Commons Attribution 4.0 International license.

In conjunction with the association of NH_4_^+^ and the C/N ratio with observed ecologic shifts, these parameters were also correlated with the abundance of one of the largest clades associated with N cycling. We found that the class *Nitrospira* was significantly correlated with both the NH_4_^+^ concentration (weighted least-squares regression, *P* = 0.039 and *F* = 4.32) and the C/N ratio (weighted least-squares regression, *P* = 0.001 and *F* = 15.71) under all tree phases within the mineral horizon (Fig. 3; Fig. S6). Specifically, as the quantity of NH_4_^+^ increased under beetle-killed trees and N became more abundant with respect to organic carbon, the relative abundance of *Nitrospira* increased.

10.1128/mBio.01305-17.6 FIG S6  The relative abundance of the *Nitrospira* class significantly correlates with the NH_4_^+^ concentration (mg of N/kg of sample dry weight) and the C/N ratio in the mineral horizon. Color indicates the extent of STM, while shape indicates whether the sample was from DNA or RNA communities. Because of the heteroscedasticity of the data, relationships were fitted by using a weighted least-squares regression where the data were weighted by the log of the independent variable (the biogeochemical variable) plus one. Download FIG S6, PDF file, 0.03 MB. Copyright © 2017 Mikkelson et al. 2017 Mikkelson et al. This content is distributed under the terms of the Creative Commons Attribution 4.0 International license.

### Exoenzyme activity.

**FIG 4:**
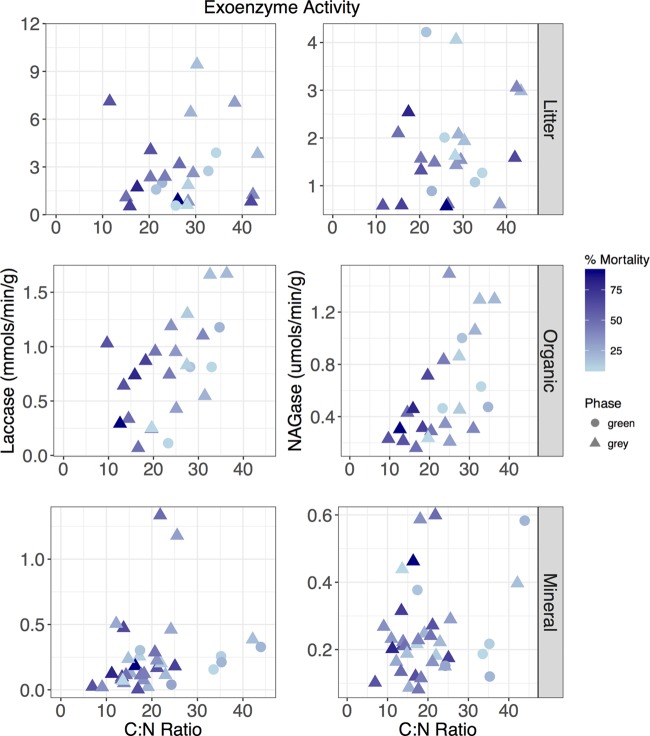
Expression of the fungal exoenzyme laccase and NAGase increases in association with a relative decrease in nitrogen bioavailability in the soil horizons beneath sampled trees. The organic horizon has greater significance when enzyme activity is assessed by using a monotonic relationship (Spearman correlations: laccase, *P* = 0.007; NAGase, *P* = 0.000), while the mineral (Pearson correlations: laccase, *P* = 0.035; NAGase, *P* = 0.032) and litter (Pearson correlations: laccase, *P* = 0.008; Nagase, *P* = 0.214) horizons have greater significance when assessed by using a linear correlation.

The activity of extracellular enzymes has been linked to nutrient availability, decay processes, and environmental conditions (25) and thus can fluctuate in perturbed ecosystems. Therefore, we investigated the activities of four microbial exoenzymes indicative of metabolic activity in forest soils. Exoenzymes indicative of more labile substrate metabolisms (*N*-acetylglucosaminidase [NAGase] and α-glucosidase) were found in greater quantities under green phase trees and smaller quantities under high-impact gray phase trees, although statistical significance was limited (Table S3). In contrast, the expression of laccase, an exoenzyme indicative of lignin depolymerization, was greater under low-impact gray phase trees than under green phase trees. It was further found in greater quantities within the litter horizon. Interestingly, significant trends were revealed when we compared enzyme activity to the C/N ratio (Fig. 4) in the litter (Pearson correlations: laccase, *P* = 0.008; NAGase, *P* = 0.214), organic (Spearman correlations: laccase, *P* = 0.007; NAGase, *P* = 0.000), and mineral (Pearson correlations: laccase, *P* = 0.035; NAGase, *P* = 0.032) horizons, suggesting an interplay between substrate bioavailability and enzyme expression. Laccase and NAGase activities both significantly increased in conjunction with the C/N ratio in every horizon, except for NAGase activity in the litter horizon.

10.1128/mBio.01305-17.9 TABLE S3  Potential enzyme activity under green phase low- and high-impact phase trees in each horizon. Reported values represent the mean ±1 standard deviation. Significant differences between tree phases in each horizon are shaded in red. Download TABLE S3, PDF file, 0.03 MB. Copyright © 2017 Mikkelson et al. 2017 Mikkelson et al. This content is distributed under the terms of the Creative Commons Attribution 4.0 International license.

## DISCUSSION

Insect-induced tree mortality in forested ecosystems has intensified globally over the past decades (3, 26). Forests play a critical role in terrestrial ecosystem services by contributing to approximately half of net global primary productivity, storing 45% of the terrestrial carbon, and exchanging greenhouse gases with the atmosphere, strongly impacting the global climate (27, 28). Large-scale insect-induced mortality in forested ecosystems has repercussions for downstream water quality (2, 11) and carbon sequestration abilities (1); however, the magnitude of these responses is not consistent across landscapes (6, 7, 9, 11, 16). Therefore, we designed a field-based sampling campaign guided by the hypothesis that observed discrepancies in forest response were the result of mitigating effects of surrounding live trees.

This is supported by prior research into logging where adjacent live trees have been shown to exert a compensatory effect and mitigate biogeochemical and hydrological impacts as a function of the clear-cut radius (29, 30). However, dynamics associated with insect-induced tree death are unique to other forest disturbances such as logging and windfall with differences in temporal decay trends and woody decay deposits (15). By analyzing both biogeochemical and microbial signatures in proximal soils at the tree scale, we were able to better understand the interplay between microscale shifts and observed biogeochemical impacts. Importantly, these terrestrial microorganisms play a large role in greenhouse gas exchange and the regulation of biogeochemical cycles (31) and therefore are a key component of ecosystem rebound after a disturbance. Results indicate that not only did soil biogeochemical responses such as respiration and C/N ratios directly correlate with the local level of STM (21), but there was a coupled response of the resident soil bacterial community beneath the beetle-killed trees.

Microbial signatures have been used as indicators of environmental perturbation, as community structure is often immediately sensitive to changes within the environment and therefore may reflect subtle shifts in ecosystem process rates (14). For instance, small variations in soil compaction during tree harvesting can create observable changes in microbial community composition and associated soil processes (22). However, as the disturbed ecosystem recovers and surrounding vegetation thrives, ecosystem process rates stabilize and can rebound. This recovery is often witnessed if the level of perturbation remains small and the microbial community contains functional redundancy. Under more extreme circumstances, the level of perturbation surpasses a threshold where these changes in the soil ecosystem become detrimental or irreversible (22). In keeping with this logic, diversity analyses of terrestrial soils within the beetle-killed forest suggest that the response of the soil microbial community is most pronounced after STM exceeds ~30 to 50% (Fig. 1 and 3). The soil microbial community beneath beetle-killed trees appears to be sensitive to even low tree mortality levels, as evidenced by the increase in alpha diversity that occurs in conjunction with increasing STM. Surprisingly, this increasing trend abruptly stops once the STM surpasses a threshold level. As alpha diversity has been associated with multifunctionality across a wide variety of terrestrial ecosystems (31), this suggests that the microbial community is able to maintain homeostasis with essential ecosystem processes such as nutrient cycling, primary production, litter decomposition, and climate regulation/feedbacks throughout a beetle infestation until the STM reaches a certain threshold. This threshold response of the microbial community may represent an indicator of larger-scale, semipermanent changes in biogeochemical cycling within beetle-killed forests. Not only did we observe significant differences at the tree scale in soil processes such as respiration and nutrient cycling under trees surrounded by >40% mortality (21), but detrimental water quality impacts have only been observed at water treatment facilities receiving water from heavily beetle-killed watersheds (2, 11).

Previous work investigating the microbial response after beetle infestation also supports this interpretation, as studies in watersheds with low tree mortality levels did not record changes in microbial community structure (9), while complementary studies in different forests with higher tree mortality levels reported significant changes in soil microbial communities and biogeochemical cycling (7, 19). By observing differences to microbial communities and soil processes under beetle-killed trees surrounded by a large spectrum of STM levels, we were able to determine that the tree mortality level within a beetle-killed watershed can explain community structure and diversity shifts within near-surface soil horizons beneath sampled trees (Fig. 1 and 3). These shifts were largest when STM surpassed a threshold of approximately 40% (Fig. 1; Fig. S4 and S5). We also found alpha diversity within the RNA community to display more prominent trends with regard to the extent of STM (Fig. 1; Fig. S2) than in the DNA community (Fig. S2 and S3). This indicates that the potentially active microbial community is more sensitive to forest disturbance and is an important contributor to the maintenance of ecosystem functionality within beetle-killed watersheds. However, significant shifts in beta diversity were still observed in both RNA and DNA community structures, despite the possibility that DNA amplicon profiles contain dead cells and free DNA bound to soil in addition to active microbes, which suggests that microbes in this environment have the ability to rapidly transition between dormancy and activity.

Interestingly, the potentially rare bacterial community (indicated by the unweighted UniFrac matrix) had the largest response to or correlation with the surrounding biogeochemical conditions. This trend is supported by a previous study in a beetle-impacted lodgepole pine forest, where results suggest that rare taxa were maintaining microbial diversity and contributing disproportionately to community dynamics and presumably biogeochemical cycling (20). It is possible that the observed shifts in the rare community composition were due to inherent heterogeneities within local soil conditions and thus the growing vegetation, as bark beetles tend to select for trees with weakened defenses in forests (32, 33); however, this linkage becomes less important as the stand mortality level increases (32). While we cannot with complete certainty say that these rare microbes are responding to tree death more strongly than their more abundant counterparts, we did control for tree diameter, species, and elevation, which have been strongly linked to the stored resources within a tree (34) and are indicative of native soil conditions. Our unique “tree-centric” approach, where all of our sampled plots were within the same watershed, also helped mitigate any possible heterogeneities that might arise when comparing plots across watersheds.

When a tree succumbs to beetle infestation, not only is there a response of the microbiota, but there are coupled changes in local hydrology and biogeochemical cycling (5, 6, 16, 21), surface water quality (2, 11), and groundwater flow dynamics (35). These changes are partially derived from differences in needle chemistry, decomposition, and leaching following beetle-induced tree death (36), combined with the cessation of rhizospheric exudates and nutrient uptake that alters the type and proportion of C and N within soil horizons. The combination of these altered inputs also helps explain why we see larger biogeochemical (21) and microbial responses in the organic and mineral horizons than in the litter horizon. Bacterial community structural shifts were most significantly associated with NH_4_^+^ concentrations and C/N ratios where trees with higher STM maintained proximal soils with higher NH_4_^+^ concentrations and lower C/N ratios than green phase trees (Fig. 3). Cladal differences between green and high-impact gray phase trees (Fig. 2) were typically associated with microorganisms whose putative functionalities are associated with carbon and nitrogen cycling, which also helps explain the observed biogeochemical shifts found under these same trees (21). The microorganisms in both RNA and DNA communities more likely to be associated with green phase trees belonged to the *Sinobacteraceae*, *Acidobacteriaceae*, *Mycobacteriaceae*, and *Burkholderiaceae* families, while those associated with high-impact gray phase trees belonged to the *C111* family. These same families were also found in greater abundances under green phase trees than under gray phase trees at a different lodgepole pine watershed where the overall tree mortality exceeded 80% (7), suggesting similar structural shifts with respect to large-scale, beetle-induced tree death despite different geographic locations within the Rocky Mountain region.

Similarly, biogeochemical signatures beneath beetle-impacted trees were also correlated with functionally relevant guilds and exoenzyme activity. The relative abundance of *Nitrospira*—a class associated functionally with nitrification (37)—was correlated with both NH_4_^+^ concentrations and C/N ratios with increased presence as the ratio of nitrogen to carbon increased (Fig. 3; Fig. S5), further indicating the intertwined nature of the changing C and N regimes beneath beetle-killed trees. Conversely, exoenzyme expression was correlated with the C/N ratio, with increased expression as carbon became more abundant with respect to nitrogen (Fig. 4). While fungal members of the community were not phylogenetically queried, NAGase has been associated with overall fungal biomass (38) and root exudation rates (39) and was significantly lower under high-impact beetle-killed trees (Table S2). This is consistent with a decrease in ectomychorrizal fungi like that seen after tree death in an analogous spruce forest (19) and the cessation of root exudates. On the other hand, laccase, an enzyme typically associated with the fungal degradation of lignin (40), had a greater net activity under both low- and high-impact beetle-killed trees than under green, healthy trees. This suggests that despite a decrease in overall fungal respiratory activity often observed in beetle-impacted forests (19), there was a shift toward increasing decay-related processes targeting recalcitrant needle deposition and woody matter decay that would dominate these beetle-impacted trees with a lesser contribution from comparatively labile root exudates excreted by surrounding live trees. In fact, a recent study investigated the carbon isotopic composition of forest soil respiration in a similar beetle-killed and girdled lodgepole pine forest and observed ^13^C-enriched soil respiration 8 to 10 years postgirdling (41). In general, the decay of coarse woody debris such as roots and tree trunks should enrich respiration, as they are more ^13^C enriched than plant leaves and sugars (42). This enrichment, similar to the exoenzyme data, indicates a shift away from the decay of more labile carbon sources and toward that of more recalcitrant sources.

Climate-induced and anthropogenic perturbations of forested ecosystems, including forest harvesting, beetle infestation, or land use changes, can have implications for greenhouse gas exchange (27), surface and groundwater quality (43), and terrestrial biogeochemical cycling (44, 45). While these larger-scale biogeochemical effects have direct implications for ecosystem and human health, the underlying smaller-scale microbial changes provide a potentially sensitive indicator of change and ecosystem resilience (14) in addition to playing an important role in these larger-scale biogeochemical processes. As soil microbial community composition can be utilized as an assessment of forest health (46), our study indicates that this might be a viable tool in beetle-killed watersheds with possible long-term utilization by forest managers, policy/decision makers, and scientists when assessing the impact of forest mortality on ecosystem function and services. While it is unknown how long the concerns associated with this type of disturbance will persist, the effects are not short-lived. Not only was this study conducted in a forest that was impacted almost a decade ago, but water quality impacts associated with disinfection by-product formation during chlorination of impacted waters have continued to increase over a decade after infestation (11). Our tree scale observations coupled with watershed scale and regional-scale studies in beetle-impacted areas (2, 10, 11) indicate that these forests harbor a resiliency capable of mitigating the effects of low STM levels that buffer ecosystem function. However, we suggest that this resiliency, as imparted by the above- and below-ground rhizospheric processes of surrounding trees along with the functional redundancy contained in the resident microbial community, has its limits in a beetle-infested forest. Specifically, once a forest surpasses a certain tree mortality level, the structure of the near-surface bacterial community, and thus the functionalities and ecosystem processes associated with an undisturbed forest, is altered. Whether or not this microscale threshold response is conserved across forests of different species and landscapes is unknown, and this study sets a foundation for the further exploration of the mechanisms and associated microbial response across various ecosystems.

## MATERIALS AND METHODS

### Site description.

This study was performed in the White River National Forest in Colorado (39.5427°N, 106.1460°W) during the summer of 2015. For full site details and sampling procedures, see reference 21. In brief, soil samples were collected under trees located in plots containing various levels of lodgepole pine (*P. contorta*) mortality within a watershed that had experienced mountain pine beetle infestation beginning in 2007-2008. Samples were collected under 7 green phase trees (i.e., healthy, transpiring trees) and 31 gray phase trees (i.e., dead trees that have ceased transpiration and dropped their needles). We defined the STM level as the number of dead trees relative to the total number of trees within an 8-m radius of the sample tree. The STM levels ranged from 9 to 91%. The plot radius was chosen as lodgepole pine roots typically extend 4 m away from the trunk (29), and thus, an 8-m radius should include trees with partially overlapping root systems and, by extension, compensatory effects on adjacent rhizospheres. Samples were further binned for selected analyses of either “high-impact” gray phase trees (trees that were surrounded by >40% tree mortality) or “low-impact” gray phase trees (all other beetle-killed trees).

### Soil sample collection and analyses.

Three composite samples were collected from the litter, organic, and mineral soil horizons under each sampled tree. Aggregate samples from each horizon were then hand homogenized into one sample per layer per tree for microbial analyses (*n* = 25 for litter, *n* = 25 for organic, *n* = 38 for mineral), with the largest focus on the mineral layer, where the largest shifts in biogeochemical parameters were observed (21). For microbial analyses, 2 g of soil was immediately collected from each bulk sample in the field and preserved in LifeGuard Soil Preservation Solution (Mo Bio Laboratories). Preserved DNA/RNA samples were stored in the dark at −80°C for <3 weeks before DNA/RNA extraction. Geochemical analyses of the homogenized bulk soil samples, including pH, soil moisture, organic matter content, dissolved organic carbon, UV absorbance at 254 nm, total nitrogen, nitrate, and ammonium, were also performed; for the methods used and the results of these analyses, see reference 21.

Four extracellular enzymes were selected for activity assays of bulk samples from each horizon. Enzyme activity potential was measured for α-glucosidase, NAGase, endo-1,4-β-glucanase (endocellulase), and laccase to encompass a suite of enzymes responsible for a hierarchy of substrate recalcitrance. Enzyme activity assays were adapted from analogous methods used in forested regions (47).

### RNA and DNA extraction.

Preserved samples for both 16S rRNA (RNA) and 16S rRNA gene (DNA) analyses were extracted with the PowerSoil Total RNA Isolation kit and the DNA Elution Accessory kit as specified by the manufacturer (Mo Bio Laboratories). Sequencing methods were adapted from references 7 and 48. Briefly, Phusion High-Fidelity DNA polymerase master mix (New England Bioscience) and nearly universal bacterial and archaeal dual indexed primers (49) were used to amplify the hypervariable V4 region. Amplicons were purified and pooled in equimolar concentrations with the SequalPrep plate normalization kit (Life Technologies, Inc.). Pooled samples were concentrated with Ultra Centrifugal filter devices (Amicon) with a 30,000 molecular weight cutoff and quantified with a Qubit 2.0 fluorometer (Life Technologies, Inc.). The library was sequenced at the BioFrontiers Institute, University of Colorado, with an Illumina MiSeq apparatus and a 2 by 250 V2 kit. Dual indexed sequencing outputs were demultiplexed by the BioFrontiers MiSeq platform.

### Microbial sequence processing.

Raw reads were joined, quality filtered, and clustered, and chimeras were removed by using the DADA2 package (50). In brief, the first 20 nucleotides were removed and sequences were trimmed at a quality score of 2. Taxonomy was assigned by using the RDP classifier v2.2 and the Greengenes v13.8 database filtered at 97% identity (51). As DADA2 does not rely on clustering, no operational taxonomic units (OTUs) were formed and we have adopted Callahan’s nomenclature referring to the nonchimeric inferred sample sequences as RSVs. RSVs not observed more than two times were removed. Sequences were also clustered into OTUs by using the more traditional USEARCH (52) algorithm with all resultant trends and findings conserved.

After the Illumina 16S rRNA and rRNA gene reads were processed and quality filtered with DADA2, a total of 2,708,333 paired-end sequences were obtained from 175 samples. Six samples with <7,000 sequences were dropped from the analysis. Sampling depths ranged from 7,826 to 42,130 sequences per sample. In addition, four outliers with low diversity were removed. After removing outliers and low-quality samples, we analyzed 22 and 24 litter samples, 25 and 22 organic samples, and 36 and 34 mineral layer samples for DNA and RNA microbial communities, respectively. All subsequent microbial analyses were based on a rarefied RSV table with 7,826 randomly selected sequences per sample, except differential abundance tests, for which a negative binomial model was used to normalize counts instead of rarefying (53).

### Downstream analyses.

Downstream analyses were completed in R (54) by using the Phyloseq (55), DESeq2 (56), and vegan (57) packages. To evaluate and visualize compositional variations as the STM changed in both DNA- and RNA-based microbial communities, we used canonical analysis of principal components (CAP) of weighted and unweighted UniFrac distance matrices (58). Adonis tests were utilized and based on the unweighted and weighted UniFrac distance matrices to quantitatively determine if the microbial communities under high-impact, low-impact, and green phase trees were different. To account for both species richness and evenness, alpha diversity was assessed by using the Shannon diversity index and RSV richness, followed by the Kruskal-Wallis and *post hoc* Dunn tests to assess significant differences in alpha diversity estimates between samples under different tree phases. Differential abundance analyses were used to identify clades that were significantly different between green and gray phase trees surrounded by either high tree mortality levels (>40%) or low tree mortality levels. For these differential abundance tests, the unrarefied RSV table was normalized and filtered with the DESeq2 package (56). Spearman and Pearson correlational tests were used to test for significant relationships between exoenzyme expression and biogeochemical parameters. The abundance of functional clades, such as *Nitrospira*, was investigated in relation to soil biogeochemical data. Because of the heteroscedasticity of the data when investigating these comparisons, relationships were fitted by using a weighted least-squares regression where the data were weighted by the log of the independent variable (the biogeochemical variable) plus one. Mantel tests were used to determine the correlation between microbial community structure and biogeochemical parameters. If necessary, *P* values were adjusted for multiple comparisons by the Bonferroni adjustment method and considered significant if less than 0.05. For the *P* values from reported statistical tests, see the associated figure legends.

### Accession number(s).

Sequences determined in this study have been deposited in the MG-RAST database under project 20837 (http://metagenomics.anl.gov/linkin.cgi?project=mgp20837).
