# Supplementary figures and images for: Ecosystem Resilience and Limitations Revealed by Soil Bacterial Community Dynamics in a Bark Beetle-Impacted Forest
Source: mBio. 2017 Dec 5;8(6):e01305-17. doi: 10.1128/mBio.01305-17 (PMC5717385; doi:10.1128/mBio.01305-17)

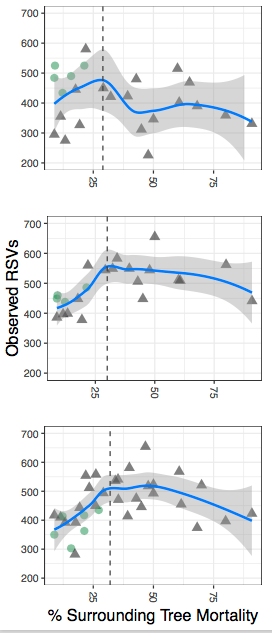

Supplement: FIG S1 [file mbo006173623sf1.tif]

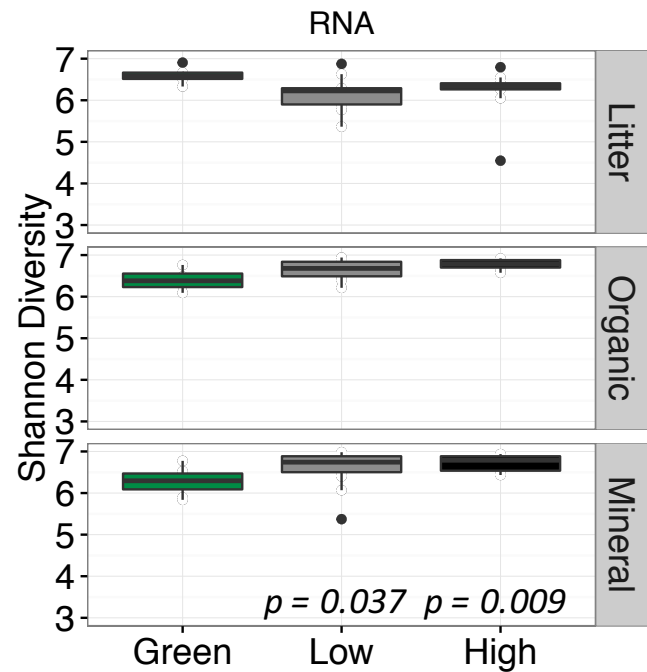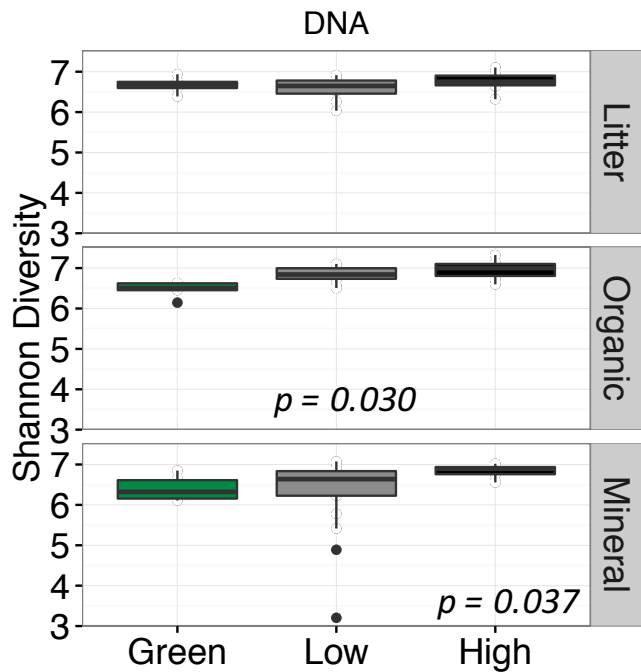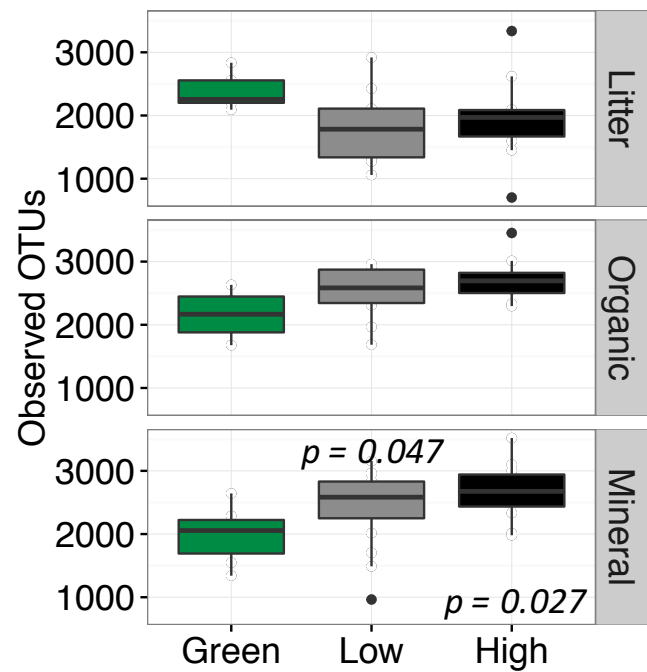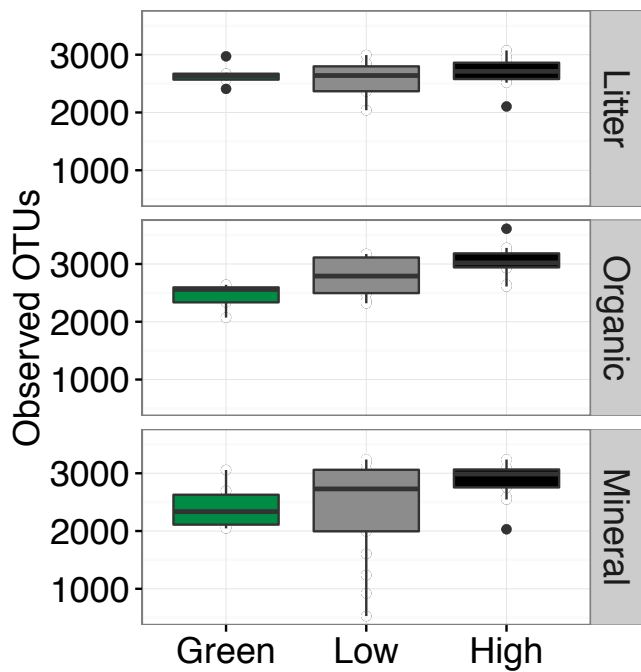

Supplement: FIG S2 [file mbo006173623sf2.pdf]

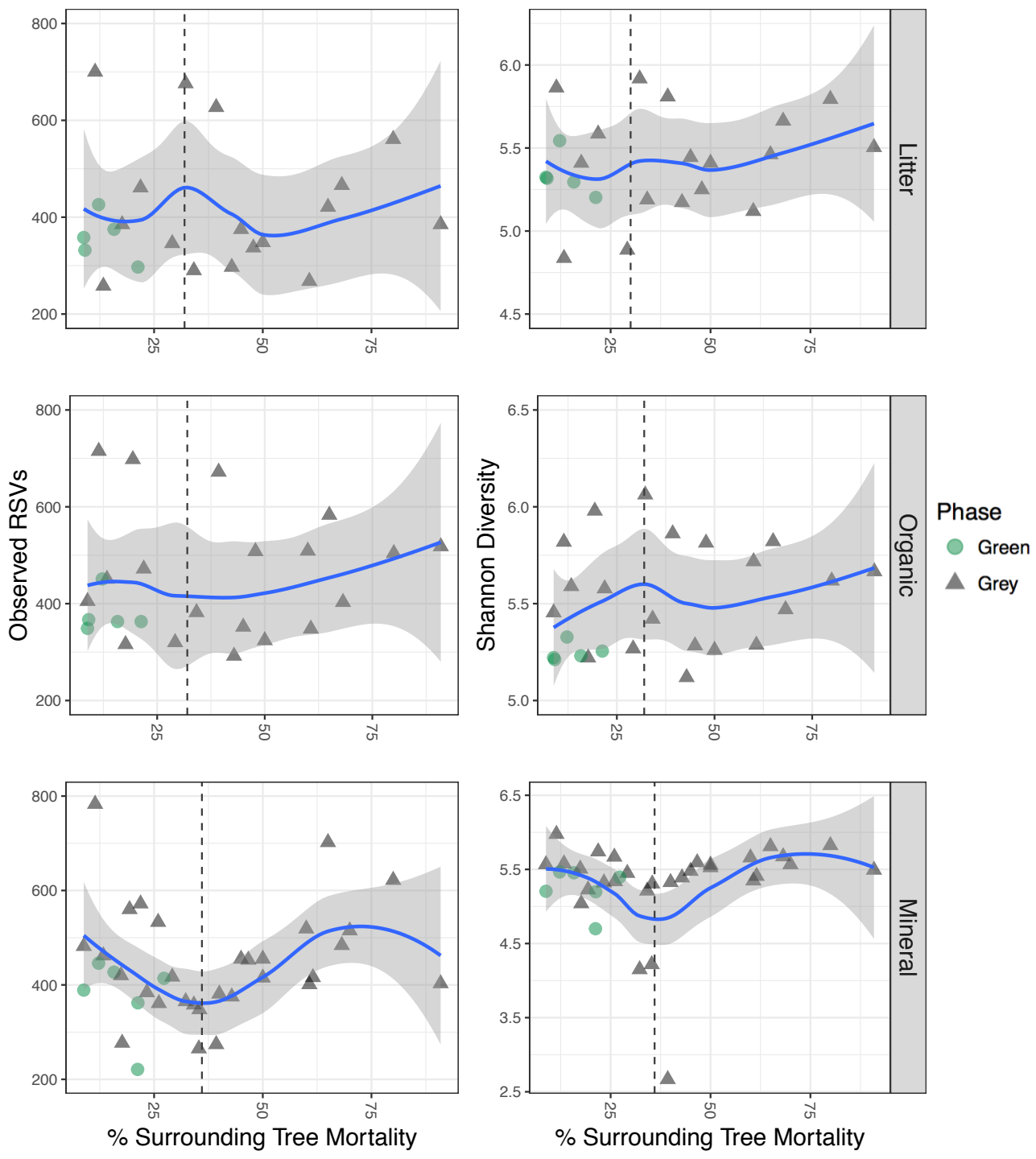

Supplement: FIG S3 [file mbo006173623sf3.pdf]

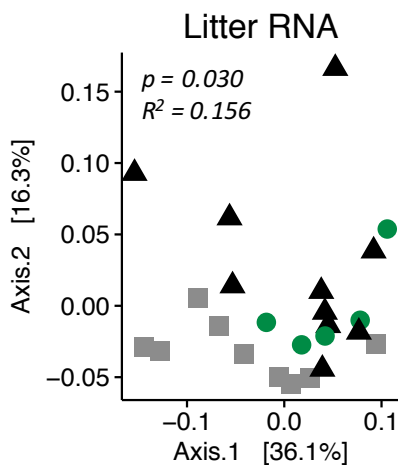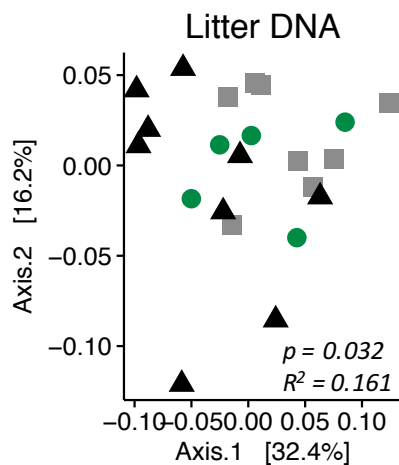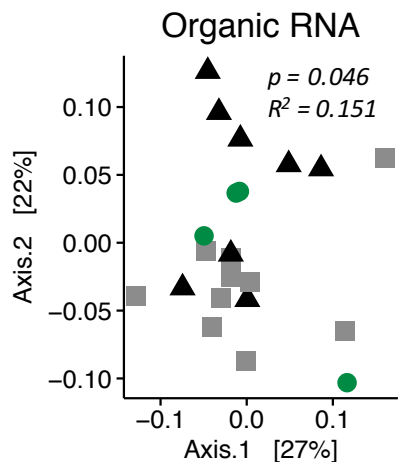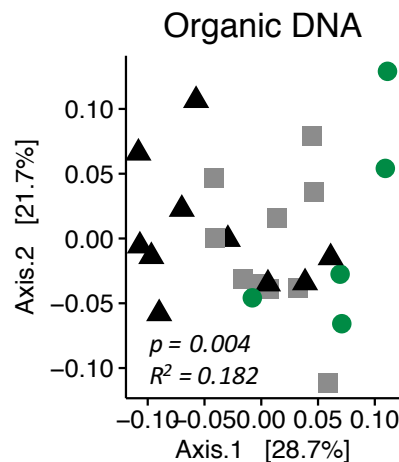

Level of Impact

- green
- low impact
- ▲ high impact

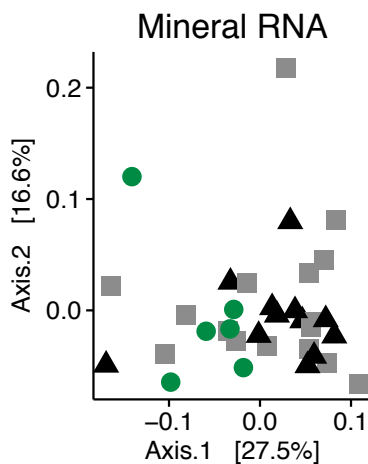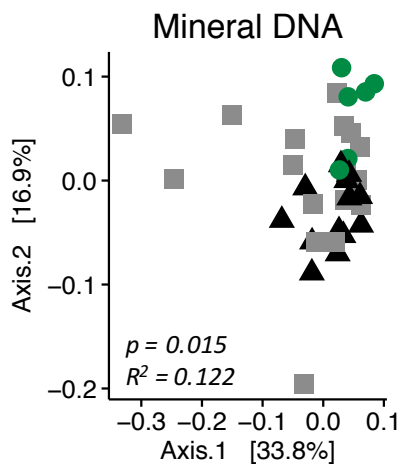

Supplement: FIG S4 [file mbo006173623sf4.pdf]

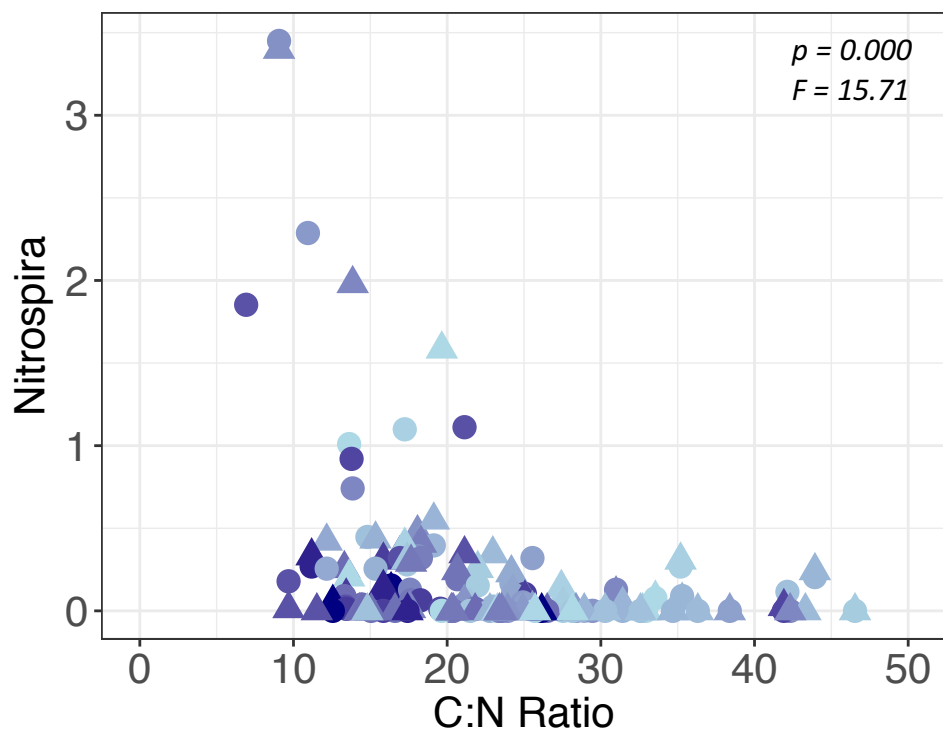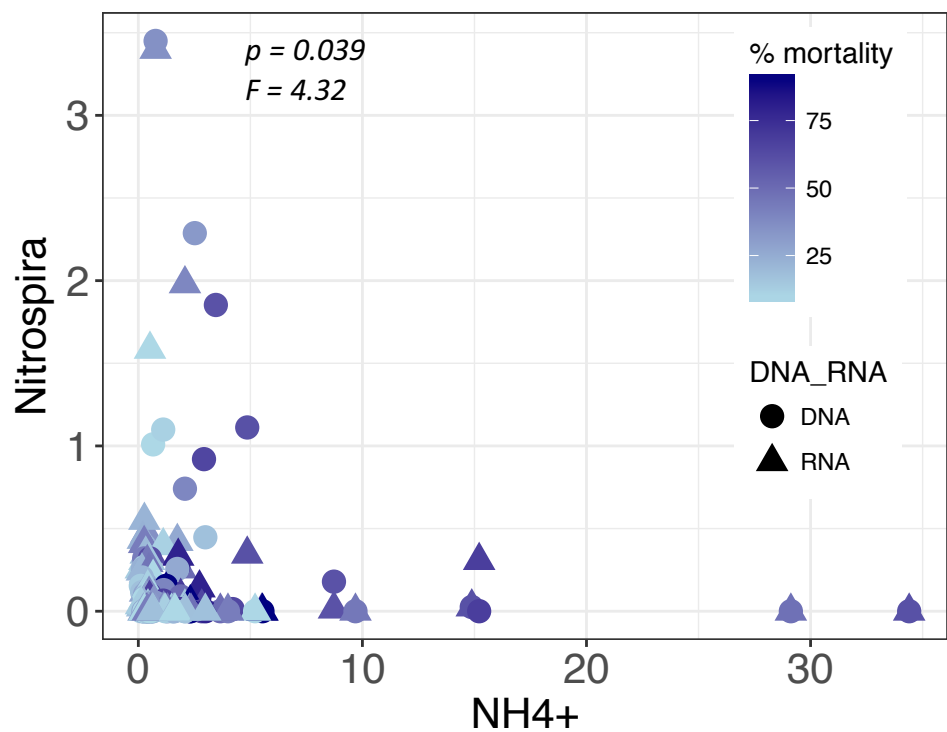

Supplement: FIG S6 [file mbo006173623sf6.pdf]
