# Supplementary material for: Ecosystem Resilience and Limitations Revealed by Soil Bacterial Community Dynamics in a Bark Beetle-Impacted Forest
Source: mBio. 2017 Dec 5;8(6):e01305-17. doi: 10.1128/mBio.01305-17 (PMC5717385; doi:10.1128/mBio.01305-17)
Supplement: FIG S5 [file mbo006173623sf5.pdf]

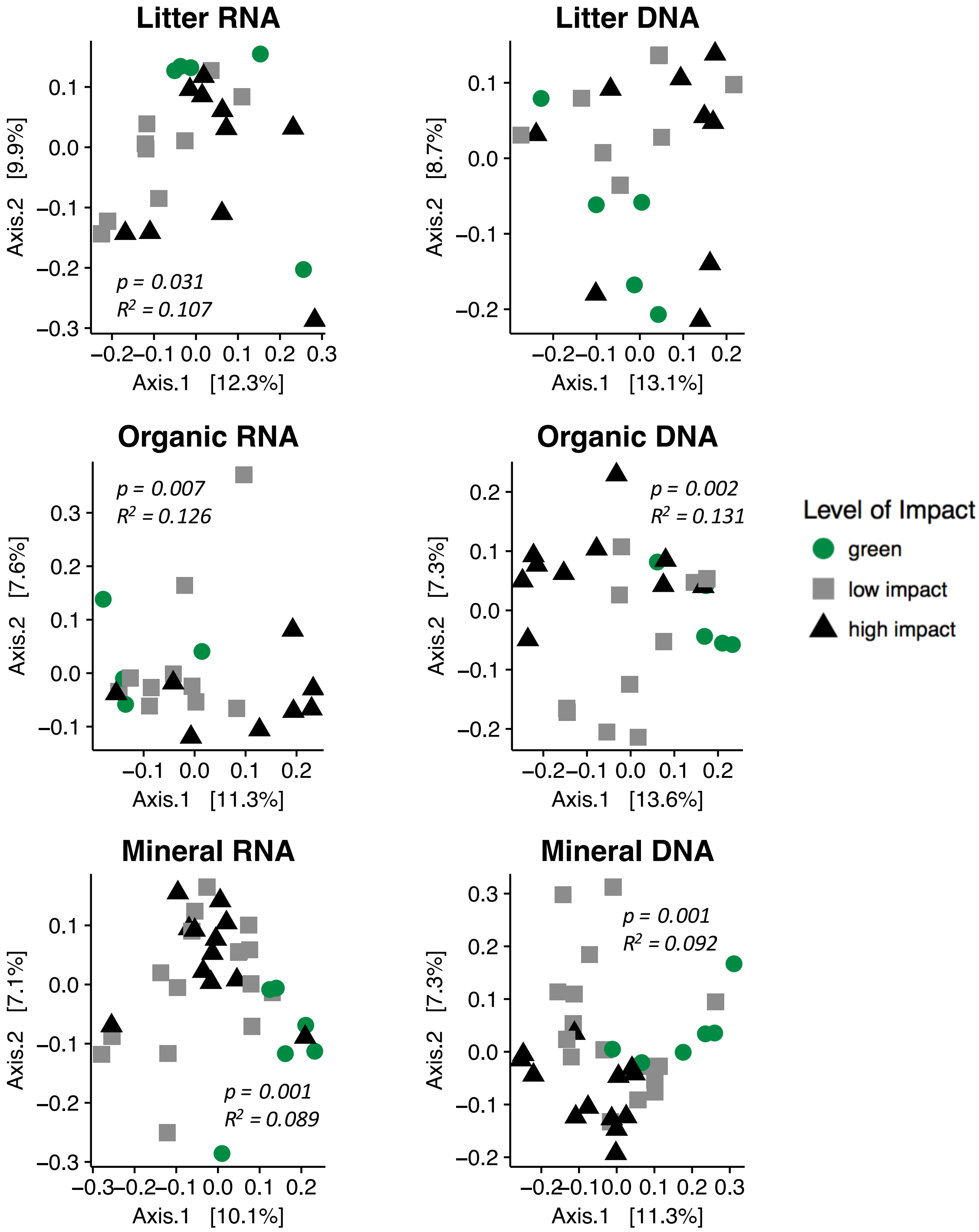

Litter RNA
p = 0.031
R2 = 0.107
Axis.2 [9.9%]
Axis.1 [12.3%]
Litter DNA
Axis.2 [8.7%]
Axis.1 [13.1%]
Organic RNA
p = 0.007
R2 = 0.126
Axis.2 [7.6%]
Axis.1 [11.3%]
Organic DNA
p = 0.002
R2 = 0.131
Axis.2 [7.3%]
Axis.1 [13.6%]
Mineral RNA
p = 0.001
R2 = 0.089
Axis.2 [7.1%]
Axis.1 [10.1%]
Mineral DNA
p = 0.001
R2 = 0.092
Axis.2 [7.3%]
Axis.1 [11.3%]
Level of Impact
green
low impact
high impact
