# Supplementary material for: Ecosystem Resilience and Limitations Revealed by Soil Bacterial Community Dynamics in a Bark Beetle-Impacted Forest
Source: mBio. 2017 Dec 5;8(6):e01305-17. doi: 10.1128/mBio.01305-17 (PMC5717385; doi:10.1128/mBio.01305-17)
Supplement: TABLE S1 [file mbo006173623st1.pdf]

Weighted UniFrac

		pH	WC	OM	DOC	SUVA	TN	NO3	NH4	C:N
DNA	Litter	-0.154	0.033	-0.105	-0.002	0.138	-0.039	0.087	0.125	0.3
	Organic	0.131	-0.207	-0.028	-0.027	0.078	0.064	0.011	0.14	0.148
	Mineral	0.449	-0.022	0.178	0.015	0.102	0.083	0.028	0.191	0.13
RNA	Litter	-0.144	-0.031	0.064	0.196	0.135	0.39	0.194	0.323	0.308
	Organic	0.059	-0.153	-0.06	-0.052	0.026	-0.014	0.013	0.059	0.075
	Mineral	0.076	0.083	-0.001	-0.135	-0.09	0.093	-0.02	0.084	-0.006

Unweighted UniFrac

		pH	WC	OM	DOC	SUVA	TN	NO3	NH4	C:N
DNA	Litter	-0.041	0.011	0.125	0.037	0.209	0.129	0.171	0.192	0.117
	Organic	0.094	-0.069	0.102	-0.157	-0.042	0.229	0.002	0.336	-0.04
	Mineral	0.392	0.029	0.366	0.279	0.13	0.133	0.106	0.245	0.407
RNA	Litter	-0.213	0.061	0.087	0.123	0.152	0.262	0.191	0.31	0.318
	Organic	-0.027	-0.09	-0.014	0.062	0.122	0.032	0.052	0.154	0.225
	Mineral	0.154	0.118	0.134	-0.021	-0.006	0.176	0.044	0.154	0.16
