# Supplementary material for: Ecosystem Resilience and Limitations Revealed by Soil Bacterial Community Dynamics in a Bark Beetle-Impacted Forest
Source: mBio. 2017 Dec 5;8(6):e01305-17. doi: 10.1128/mBio.01305-17 (PMC5717385; doi:10.1128/mBio.01305-17)
Supplement: TABLE S2 [file mbo006173623st2.pdf]

A

		pH	WC	OM	DOC	SUVA	TN	NO3	NH4	C:N
DNA	Litter	-0.169	0.049	0.101	-0.058	0.1464	0.084	0.033	0.239	0.156
	Organic	-0.03	-0.12	0.012	0.044	0.038	0.204	0.022	0.175	0.091
	Mineral	0.377	-0.073	0.358	0.298	0.087	0.171	0.184	0.182	0.345
RNA	Litter	-0.188	-0.022	0.039	0.02	0.054	0.463	0.079	0.501	0.061
	Organic	0.03	-0.177	-0.029	-0.068	0.062	0.033	-0.037	0.065	0.058
	Mineral	0.088	0.091	-0.027	-0.078	-0.07	0.064	0	0.098	0.027

B

		pH	WC	OM	DOC	SUVA	TN	NO3	NH4	C:N
DNA	Litter	-0.184	0.025	-0.124	0.079	0.026	-0.059	0.072	0.116	0.348
	Organic	0.086	-0.216	-0.016	-0.041	0.108	0.097	0	0.164	0.155
	Mineral	0.377	-0.073	0.358	0.298	0.087	0.171	0.184	0.181	0.345
RNA	Litter	-0.112	0.014	-0.004	0.353	0.238	0.305	0.316	0.197	0.34
	Organic	0.177	-0.112	-0.061	-0.012	0.037	-0.024	0.024	0.029	0.096
	Mineral	0.088	0.091	-0.027	-0.078	-0.069	0.064	0	0.202	0.027
