# Supplementary material for: Ecosystem Resilience and Limitations Revealed by Soil Bacterial Community Dynamics in a Bark Beetle-Impacted Forest
Source: mBio. 2017 Dec 5;8(6):e01305-17. doi: 10.1128/mBio.01305-17 (PMC5717385; doi:10.1128/mBio.01305-17)
Supplement: TABLE S3 [file mbo006173623st3.pdf]

		Enzyme Activity Potential			
		α-glucosidase	NAGase	endocellulase	laccase
Litter	Green	0.29 ± 0.20	1.90 ± 1.30	0.07 ± 0.04	2.16 ± 1.18
	Low	0.31 ± 0.27	2.22 ± 0.99	0.08 ± 0.06	4.53 ± 3.74
	High	0.22 ± 0.16	1.30 ± 0.70	0.05 ± 0.03	2.42 ± 1.95
Organic	Green	0.11 ± 0.06	0.66 ± 0.22	0.02 ± 0.00	0.62 ± 0.43
	Low	0.08 ± 0.04	0.76 ± 0.50	0.02 ± 0.01	0.99 ± 0.48
	High	0.11 ± 0.06	0.40 ± 0.22	0.02 ±0.01	0.58 ± 0.33
Mineral	Green	0.20 ± 0.10	0.25 ± 0.16	0.02 ± 0.01	0.19 ± 0.12
	Low	0.06 ± 0.01	0.25 ± 0.12	0.02 ± 0.01	0.25 ± 0.28
	High	0.08 ± 0.05	0.23 ± 0.14	0.03 ± 0.01	0.24 ± 0.33
